# Supplementary material for: Sexualized Drug Use and Chemsex among Men Who Have Sex with Men in Europe: A Systematic Review and Meta-Analysis
Source: J Clin Med. 2024 Mar 21;13(6):1812. doi: 10.3390/jcm13061812 (PMC10971601; doi:10.3390/jcm13061812)
Supplement: Supplementary file 1 [file jcm-13-01812-s001.zip › Supplementary Material S1.pdf]

Supplemental Material S1: Search strategy

DATABASE	RESEARCH STRAGEGY	FILTER	DOCUMENTS
**PubMed**	**MeSH:** (“Substance-Related Disorders”[MeSH Terms] AND “Unsafe Sex”[MeSH Terms]) AND “Sexually Transmitted Diseases”[MeSH Terms] AND (“Homosexuality, Male”[MeSH Terms] OR “Sexual and Gender Minorities” [MeSH Terms])	**Published during the last 6 years.**	**91**
	**Free search:** (“Chemsex” OR (“Substance-Related Disorders” OR “Drug Abuse” OR “Drug Addiction” OR “Drug Dependence” OR “Drug Habituation” OR “Drug Use Disorder*” OR “Substance Abuse*” OR “Substance Addiction” OR “Substance Dependence” OR “Substance RelatedDisorder” OR “Substance Use*” OR “Substance Use Disorder*” OR “Nitrite*”) AND (“Condomless Sex” OR “High Risk Sex” OR “High-Risk Sex” OR “Unprotected Intercourse” OR “Unprotected Sex” OR “Unsafe Sex”)) AND (“Sexually Transmitted Disease*” OR “Sexually Transmitted Infection*” OR STD OR STDs OR STI OR STIs OR “Venereal Disease*” OR “Sexually Transmitted Diseases”) AND (“Homosexuality, Male” OR “Sexual and Gender Minorities” OR “Men Who Have Sex With Men” OR “Gay” OR “Gays”)		**181**
**SCOPUS**	(“Chemsex” OR (“Substance-Related Disorders” OR “Drug Abuse” OR “Drug Addiction” OR “Drug Dependence” OR “Drug Habituation” OR “Drug Use Disorder*” OR “Substance Abuse*” OR “Substance Addiction” OR “Substance Dependence” OR “Substance Related Disorder” OR “Substance Use*” OR “Substance Use Disorder*” OR “Nitrite*”) AND (“Condomless Sex” OR “High Risk Sex” OR“High-Risk Sex” OR “Unprotected Intercourse” OR “Unprotected Sex” OR “Unsafe Sex”)) AND (“Sexually Transmitted Disease*” OR “Sexually Transmitted Infection*” OR STD OR STDs OR STI OR STIs OR “Venereal Disease*” OR “Sexually Transmitted Diseases”) AND (“Homosexuality,Male” OR “Sexual and Gender Minorities” OR “Men Who Have Sex With Men” OR “Gay” OR “Gays”)		**115**
**Cochrane Library**	(“Chemsex” OR (“Substance-Related Disorders” OR “Drug Abuse” OR “Drug Addiction” OR “Drug Dependence” OR “Drug Habituation” OR “Drug Use Disorder*” OR “Substance Abuse*” OR “Substance Addiction” OR “Substance Dependence” OR “Substance Related Disorder” OR “Substance Use*” OR “Substance Use Disorder*” OR “Nitrite*”) AND (“Condomless Sex” OR “High Risk Sex” OR “High-Risk Sex” OR “Unprotected Intercourse” OR “Unprotected Sex” OR “Unsafe Sex”)) AND (“Sexually Transmitted Disease*” OR “Sexually Transmitted Infection*” OR STD OR STDs OR STI OR STIs OR “Venereal Disease*” OR “Sexually Transmitted Diseases”) AND (“Homosexuality, Male” OR “Sexual and Gender Minorities” OR “Men Who Have Sex With Men” OR “Gay” OR “Gays”)		**6**
**Embase**	**Entree:** ('chemsex' OR ('unsafe sex' AND 'drug dependence')) AND 'sexually transmitted disease' AND ('men who have sexwith men' OR 'homosexual male')		**145**
	**PICO search:** (('unsafe sex' OR 'high-risk sex' OR 'high-risk sexual behavior' OR 'high-risk sexual behaviour') AND ('addiction, drug' OR 'dependence, drug' OR 'drug addict' OR 'drug addiction' OR 'drug dependence model' OR 'drug dependency' OR 'drug habituation' OR 'drug physical dependence' OR 'substance addiction' OR 'substance dependence' OR 'substance dependency' OR 'substance use disorder' OR 'substance use disorders' OR 'substance-related disorder' OR 'substance-related disorders' OR 'drug dependence' OR 'nitrite') OR 'chemsex') AND ('bacterial sexually transmitted diseases' OR 'sexually transmitted disease'OR 'sexually transmitted diseases' OR 'sexually transmitted diseases, bacterial' OR 'sexually transmitted diseases, viral' OR'sexually transmitted infection' OR 'std' OR 'vd' OR 'veneral disease' OR 'venereal disease' OR 'venereal infection' OR 'viralsexually transmitted diseases') AND ('men who have sex with men' OR 'homosexual male' OR 'gays')		**170**

DATABASE	RESEARCH STRAGEGY	FILTER	DOCUMENTS
**Web of Science (WoS)**	((“Substance-Related Disorders”[MeSH Terms] AND “Unsafe Sex”[MeSH Terms]) AND “Sexually Transmitted Diseases”[MeSH Terms] AND (“Homosexuality, Male”[MeSH Terms] OR “Sexual and Gender Minorities” [MeSH Terms]) OR (“Chemsex” OR (“Substance-Related Disorders” OR “Drug Abuse” OR “Drug Addiction” OR “Drug Dependence” OR “Drug Habituation” OR “Drug Use Disorder*” OR “Substance Abuse*” OR “Substance Addiction” OR “SubstanceDependence” OR “Substance Related Disorder” OR “Substance Use*” OR “Substance Use Disorder*” OR “Nitrite*”) AND (“Condomless Sex” OR “High Risk Sex” OR “High-Risk Sex” OR “Unprotected Intercourse” OR “Unprotected Sex” OR “Unsafe Sex”)) AND (“Sexually Transmitted Disease*” OR “Sexually Transmitted Infection*” OR STD OR STDs OR STI OR STIs OR “Venereal Disease*” OR “Sexually Transmitted Diseases”) AND (“Homosexuality, Male” OR “Sexual and Gender Minorities” OR “Men Who Have Sex With Men” OR “Gay” OR “Gays”))		**197**
