# Supplementary material for: Sexualized Drug Use and Chemsex among Men Who Have Sex with Men in Europe: A Systematic Review and Meta-Analysis
Source: J Clin Med. 2024 Mar 21;13(6):1812. doi: 10.3390/jcm13061812 (PMC10971601; doi:10.3390/jcm13061812)
Supplement: Supplementary file 1 [file jcm-13-01812-s001.zip › Supplementary Material Table S1.pdf]

Table T1. Summary of selected manuscripts.

TITTLE, AUTHOR, PUBLICATION YEAR AND COUNTRY	STUDY, QUALITY AND OBJECTIVES	METHODS	RESULTS	CONCLUSIONS
**Prevalence of drug use during sex amongst MSM in Europe: Results from a multi-site bio-behavioural survey [28]**<br><br>Rosisnka et al 2018<br>Countries (ω)	Cross-sectional study.<br><br>STROBE:14/22<br><br>Examine the frequency and determinants of substance consumption during sexual encounters and discern distinct requirements for prevention measures.	Survey conducted between 2013 and 2014 Data were collected through direct interview. Oral liquid and blood sample were also collected.<br><br>Sample size (N): 1260	✓ A total of 142 (3.4%) use chemsex drugs.<br><br>✓ The use of all the analyzed substances was significantly associated with sexual encounter with more than one partner.<br><br>✓ Individuals who reported drug use were notably more likely to be diagnosed with HIV both before (10.5% vs. 3.9%) and with other sexually transmitted infections (STIs) during the 12-month period preceding the study (16.7% vs. 9.2%).	Substance and polydrug utilization during sexual engagements were evident among MSM across Europe, with notable disparities. Diverse social norms may significantly influence drug consumption patterns.
**Chemsex, risk behaviours and sexually transmitted infections among men who have sex with men in Dublin, Ireland [29]**<br><br>Glym et al 2018<br>Ireland	Cross-sectional study.<br><br>STROBE: 14/22<br><br>Assessing the prevalence of chemsex, associated behaviors and STIs	Study conducted in 2016 in Dublin in patients attending the Gay Men's Health Service (GMHS). Sample size (N) 568	✓ The prevalence of chemsex use were 27%<br><br>✓ Individuals participating in chemsex exhibited a higher propensity for engaging in sexual activity with multiple partners, particularly for anal intercourse, and were more inclined to engage in condomless anal intercourse.<br><br>✓ A higher likelihood of reporting treatment for gonorrhea within the preceding 12 months.	A comprehensive approach is necessary to address the intersecting issues of addiction and sexual health among MSM who encounter harm or seek assistance due to involvement in chemsex.

TITTLE, AUTHOR, PUBLICATION YEAR AND COUNTRY	STUDY, QUALITY AND OBJECTIVES	METHODS	RESULTS	CONCLUSIONS
**Chemsex among men who have sexwith men living outside major cities and associations with sexually transmitted infections: A cross-sectional study in the Netherland [30]**<br><br>Evers Y.J et al. 2019. The Netherlands	Cross-sectional study.<br><br>STROBE:18/22<br><br>Assessing the prevalence of chemsex and other patterns of risky sexual behavior in MSM.<br><br>Studying associations with STIs in regions surrounding major cities.	Study conducted between 2017 and 2018 in STI clinics in the Netherlands. Data were collected through an online questionnaire on drug use among MSM. Additionally, various STI tests were administered.<br><br>Sample size (N): 250	✓ A total of 54% of the population reported SDU. The most used substances were 'poppers,' MDMA/ecstasy, and GHB/GBL. Approximately 35% of the population engaged in 'chemsex.' A comparison of "chemsex" prevalence revealed 36% among urban MSM and 33% among nonurban MSM.<br><br>✓ CAI was reported by 64% of the participants.<br><br>✓ A total of 19% of the participants tested positive for STIs. The association between "chemsex" and STIs was similar, albeit slightly lower outside major urban areas.	Positive diagnoses of STIs are associated with the concurrent use of multiple drugs among the MSM community.<br><br>The use of substances during sexual encounters is similar among MSMs residing outside major cities in the Netherlands. The prevalence of "chemsex" exhibits a close resemblance, albeit slightly reduced, in nonurban regions.
**Sex, drugs, and sexually transmitted infections: A latentclass analysis among men who have sex with men in Amsterdam and surrounding urban regions, the Netherlands. [31]**<br><br>Achterbergh R.C.A et al.	Cross-sectional study.<br><br>STROBE:17/22.<br><br>Investigate whether there are differences in SDU among HSH in Amsterdam and nearby urban areas, assessing patterns, risky behaviors, and	Study conducted between September and December 2017 in Amsterdam and surrounding regions, involving HSH attending sexual health clinics for three consecutive months.	✓ A total of 54.8% of the population reported SDU, with a higher prevalence of consumption in Amsterdam compared to the urban outskirts.<br><br>✓ Different patterns of drug use were observed between Amsterdam and nearby areas. In Amsterdam, there are four types ranging from non-consumption to high usage, while in the surrounding areas, there are three groups ranging from non-consumption to a broad	Significant differences are identified in the pattern of SDU between Amsterdam and urban areas in the vicinity, with the use of 'polydrugs' exhibiting the highest prevalence of STI among all groups.

TITTLE, AUTHOR, PUBLICATION YEAR AND COUNTRY	STUDY, QUALITY AND OBJECTIVES	METHODS	RESULTS	CONCLUSIONS
2020, The Netherlands	STIs.	Sample size (N): 4461.	range of substance use.<br>✓ The 'polydrug' group showed a higher prevalence of risky behaviors and STI in both areas.	
**Patterns of sexualised recreational druguse and its association with risk behaviours and sexual health outcomes in men who have sex with men in London, UK: a comparison of cross-sectional studies conducted in 2013 and 2016 [32]**<br>Curtis T.J et al. 2020, United Kingdon.	Cross-sectional study.<br>STROBE:16/22<br>Study the patterns of SDU and their association with risky sexual behaviors, STI diagnoses, and healthcare-seeking behavior in the HSH population.	Study performed using a self-reported questionnaire in the HSH population in London during 2013 and 2016.<br>Samples from 2013, N=905; and 2016 N= 739 subjects.	✓ The prevalence of "chemsex" practice did not vary between the years 2013 and 2016. The most consumed "chemsex" drug in both years was mephedrone.<br>✓ Subjects who reported engaging in "chemsex" were more likely to have or participate in sexual relationships considered risky.	The prevalence of "chemsex" remained unchanged between 2013 and 2016, indicating that it continued to be high. Chemsex was associated with participation in risky behaviors. The frequency of HIV testing was lower in the last year compared to the national recommendations in London.
**Association of sexualized drug use patterns with HIV/STI transmission risk in an-internet sample of**	Cross-sectional study.<br>STROBE:19/22<br>Identify the patterns	Study conducted between April and December 2016 in 7 European countries.	✓ The two most predominant SDU patterns were 'solely drugs for enhancing sexual performance' and 'party drugs, but not for chemsex.' The most common substance used for enhancing sexual	A lower prevalence SDU is observed compared to other countries, despite higher rates of STIs/HIV.

TITTLE, AUTHOR, PUBLICATION YEAR AND COUNTRY	STUDY, QUALITY AND OBJECTIVES	METHODS	RESULTS	CONCLUSIONS
**men whohave sex with men from seven european countries [33]**<br><br>Guerras J.M et al. 2020, ψ	of SDU. Determine the sexual risk profiles associated with each pattern and characterize the individuals involved in high-risk sexual behavior among MSM.	The survey assesses sociodemographic factors, external factors, testing history, serological status, and risky sexual behaviors, among other parameters.<br><br>Sample size (N): 9407.	performance within this pattern was 'poppers.'<br><br>✓ Participants who engaged in SDU significantly differed from nondrug users in sexual contexts and behaviors.<br><br>✓ Among SDU consumers, a higher prevalence of HIV and other STIs was observed.<br><br>✓ Diagnoses of HIV/STIs and risky sexual behaviors increased from non-SDU to 'chemsex drugs,' and the combination of these with other substances, especially party drugs.	In certain countries, a higher prevalence of SDU is observed, particularly among MSMs residing in major urban areas.
**Sexual behavior and drug use impact in gay, bisexual, and othermen who have sex withmen [34]**<br><br>García-Pérez J.Net al. 2022, Spain.	Cross-sectional study<br><br>STROBE:18/22.<br><br>Analyzing 'chemsex' and STIs in GBMSM population<br><br>Comparing drug-using population with non-using population.	Data extracted from a sexual health clinic in Barcelona between January 2019 and July 2020 through an anonymous, self-administered survey.<br><br>Sample size N=514	✓ A total of 26.5% used chemsex at least once in the last year. GHB was the drug most used.<br><br>✓ Number of sexual partners, group sex sessions, and CAI/CAR were more frequent among chemsex users.<br><br>✓ HIV, prevalence was twice as high in chemsex users, and had more STIs than non-users	In the GBMSM population, the use of chemsex is widespread and is associated with group sex, HIV, and STIs.

TITTLE, AUTHOR, PUBLICATION YEAR AND COUNTRY	STUDY, QUALITY AND OBJECTIVES	METHODS	RESULTS	CONCLUSIONS
**Substance use in sexual context among Spanish resident men who have sex with men [35]**<br><br>Guerras J.M et al. 2022, φ	Cross-sectional study<br><br>STROBE:17/22.<br><br>Identifying SDU patterns in MSM and describing the prevalence of risky sexual behaviours, HIV, and STIs.	Online survey carried out between April and December 2016.<br><br>Sample size N=2883	✓ The occurrence rate of SDU stood at 21.9%. The nitrile derivative "poppers" emerged as the most utilized, with GHB/GBL being the primary choice among chemsex users.<br><br>✓ Chemsex was associated with CAI, paid for sex and group sex session.<br><br>✓ Chemsex was more prevalent in HIV+ (21,9%) vs HIV- (6,6%).	The user of the chemsex, it might contribute to the transmission of STIs. .

ω Belgium, Bulgaria, Germany, Italy, Lithuania, Poland, Portugal, Romania, Slovakia, Slovenia, Spain, Sweden, and United Kingdom; φ Belgium, Denmark, Grece, Germany, Portugal, Romania, Slovenia and Spain; STI, sexually transmitted infection; MSM, Men who have Sex with Men; SDU sexualized drugs user CAI, Condomless Anal Intercourse; CAR, Condomless Anal Receptive; HIV; Human immunodeficiency virus; GHB/GBL gamma-hydroxybutyrate/gamma-butyrolactone.
